# Cancer burden in the Caribbean: an overview of the Martinique Cancer Registry profile

**DOI:** 10.1186/s12885-019-5434-6

**Published:** 2019-03-15

**Authors:** Clarisse Joachim, Jacqueline Veronique-Baudin, Stephen Ulric-Gervaise, Audrey Pomier, Aimée Pierre-Louis, Mylène Vestris, Jean-Luc Novella, Moustapha Drame, Jonathan Macni, Patrick Escarmant

**Affiliations:** 1 CHU Martinique, UF1441 Registre des cancers de la Martinique, Pôle de Cancérologie Hématologie Urologie Pathologie, Fort-de-France, Martinique; 2 Association Martiniquaise pour la Recherche en Cancérologie en Martinique, Registre Général des cancers de la Martinique, Fort-de-France, Martinique; 3 CHU de Reims, Pôle de Gériatrie, Reims, France; 4 CHU de Martinique, UF 3163, Unité de soutien méthodologique à la Recherche, Délégation de la Recherche et de l’innovation, Fort-de-France, Martinique; 5 CHU Martinique, Pôle de Cancérologie Hématologie, Urologie Pathologie, CHU Martinique, Fort-de-France, Martinique

**Keywords:** Cancer, Epidemiology, Surveillance, Caribbean, Registry

## Abstract

**Background:**

Cancer indicators are essential information for cancer surveillance and cancer research strategy development. The Martinique Cancer Registry (MCR) is a population-based cancer Registry (PBCR) that has been recording cancer data since its creation in 1981. This article provides cancer incidence and mortality data for all cancers and for major tumor sites.

**Methods:**

The registry collects all new cancer cases, details of the individual affected, tumor site and follow-up. World-standardized incidence and mortality rates were calculated, by tumor site and sex for solid tumors from the MCR database for the study period 2001–2015.

**Results:**

Over the period 2001–2015, a total of 22,801 new cases were diagnosed; 13,863 in men (60.8%) and 8938 in women (39.2%). In 2011–2015, 1631 new cases were diagnosed per year. Age-standardized (to the world population) incidence rates for all cancers, were 289.8 per 100,000 men and 171.0 per 100,000 women. Breast, colon-rectum and stomach were the most common cancer sites in women. Prostate, colon-rectum and stomach were the main sites in men. Martinique has higher incidence rates of prostate and stomach cancer than mainland France.

**Conclusions:**

Prostate and stomach cancers have high incidence and rank first among the four major tumor sites. Providing data for the French zone of the Caribbean is essential to contributing to the development of high-priority public health measures for the Caribbean zone.

## Background

In 2012, 14.1 million new cancer cases and 8.2 million cancer deaths occurred worldwide, and 32.5 million people were living with a cancer diagnosed in the previous 5 years [1]. Almost 1.1 million new cancer cases were estimated in Latin America and the Caribbean, with a total number of deaths estimated at around 600,000 [2].

Health indicators generated by population-based cancer registries (PBCR) are useful not only for the population, but also for researchers, clinicians, healthcare establishments and local authorities, as well as public health decision-makers. Projections for the cancer burden up to 2030, based on PBCR data in this region, highlight that cancer will become increasingly prevalent in the future, to reach an estimated 1.6 million new cancer cases by 2030 [2].

Cancers in the Caribbean countries were more frequently infection-related cancers (cervical cancer, stomach…), but the cancer profile is changing. Overall, prostate, colorectal and breast cancer are now the leading causes of cancer in the Caribbean, with higher rates of prostate cancer (PCa) incidence and mortality among black populations in this region. Geographic variations in PCa exist, with the highest rates observed in the French Caribbean West Indies (Martinique, Guadeloupe) [3]. Changes in lifestyle factors, such as decreased fertility and birth rates, changes in tobacco smoking habits, and changes in diet, most likely explain the changing cancer profile in the Caribbean. Differences in diagnostic and treatment practices, access to healthcare and public health policy, could further explain the overall variations observed in the Caribbean.

Stomach and cervical cancers continue to represent a major public health challenge to reducing the burden of cancer in this region. Despite the decreasing mortality rates of stomach cancer observed worldwide [4], this cancer continues to rank among those with the highest incidence and mortality rates in the Caribbean. Major research into these cancers has been designated as a national priority, via collaboration and research with PBCRs in the Caribbean. These themes are integral components of the French national cancer plan for the 2014–2019 period.

The Martinique Cancer Registry (MCR) is pursuing a national policy in terms of public health and research within the Caribbean. These public health missions enabled the Registry, in accordance with current and future plans for public health within the national cancer plan for the period 2014–2019, to produce reference indicators to describe the dynamics of cancer trends, to evaluate the state of health of the population, and to identify the main factors associated with the occurrence of this disease, its deterioration, or the extent of its repercussions. Analysing management patterns in patients with cancer, taking into account vulnerable populations such as elderly patients with cancer, or young patients, is also one of the main research priorities of the MCR. Identifying populations at risk and helping to reduce inequalities among cancer patients is a major challenge for the Caribbean. In this study, we describe the incidence and mortality rates in Martinique, for all cancers and major sites.

## Methods

### Population and design

This retrospective population-based study included all incident patients with invasive cancer diagnosed in Martinique between 01/01/2001 and 12/31/2015. Data were recorded in the MCR in strict conformity with the international standards laid down by the International Agency for Research on Cancer [5], the French FRANCIM network, and the European Network of Cancer Registries (ENCR). Registry procedures were approved by the French National authority for the protection of privacy and personal data.

### Data collection

Thanks to data cross-matching and analysis of all available data sources, the MCR guarantees high quality information about cancer in the region of Martinique. The registry is currently actively cooperating with a range of local organisations to ensure an exhaustive data collection circuit (discharge reports, laboratory results, pathology findings, people qualified as having chronic disease by the social security, clinical patient files…). Data were extracted anonymously using the International Classification of Diseases version 10 –[6] codes and the International Classification of Diseases for Oncology - Third version [7]. Mortality data were obtained from the French epidemiological centre on medical causes of death – French National Institute of Health and Medical Research (CépiDc, Inserm: https://www.cepidc.inserm.fr/). Data from the year 2012 were not included, due to incompleteness of French national data for this year [8].

### Statistical analysis

For study purposes, we considered mortality data for all cancers and for major cancer sites: prostate, female breast, colon, rectum, stomach, and lung and bronchial cancers. The Segi-Doll world standard was used to calculate world-standardized incidence and mortality rates, by 5-year periods to enable comparisons of cancer risk between registries, independently of the effects of age [9, 10]. Direct standardization computes the weighted average of stratum-specific estimates in the study population, using the weights from a standard or reference population.

Annual rate of change (ARC) was calculated using Poisson regression and is presented as a percentage with 95% confidence interval (CI) for the 2001–2015 period and by 5-year period. Due to the incompleteness of 2012 mortality data the ARC was not estimated for mortality. Incidence and mortality rates were plotted for individual years, except for 2012, due to the incompleteness of 2012 mortality data. We looked at all possible segments of the data by 5-year period and reported in the Results and in the Figures, those that were significant (statistically). Cumulative rates up to the age of 74 years were also presented. All analyses were performed using SAS version 9.4. (SAS Institute Inc., Cary, NC, USA).

## Results

### All cancers

For the period 2001–2015, 22,801 new cases of cancer occurred (excepted skin carcinoma) in Martinique, 13,863 in men (60.8%) and 8938 in women (39.2%). A mean of 1631 new cancer cases per year were recorded by the MCR over the last period 2011–2015.

**Fig. 1:**
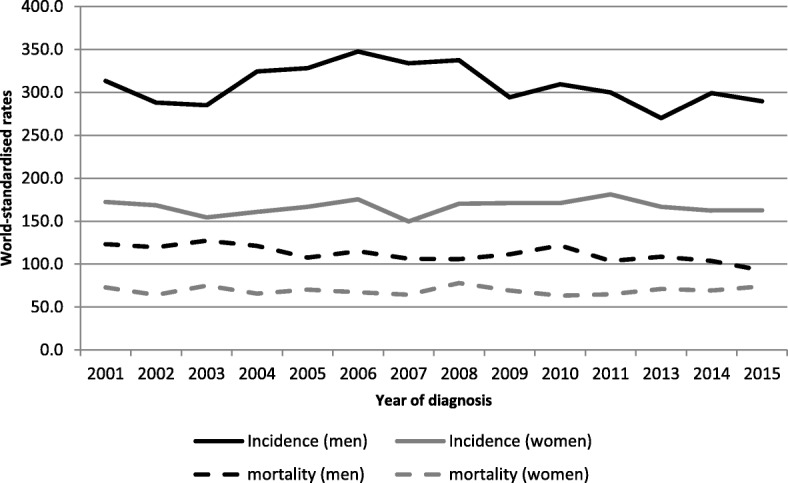
Incidence and mortality world-standardised rates, by year of diagnosis, in all cancers, Martinique, 2001–2015. Footnotes: Significant ARC for incidence by 5-year period in men: 2.75 (2001–2005); − 3.97 (2006–2010). ARC in women: − 3.01 (2011–2015).

Figure 1 presents world-standardised incidence and mortality rates, by year of diagnosis in all cancers during the study period.

Four sites account for more than half of new cancer cases for both sexes (*n* = 14,195; 62.3% of cases): prostate, breast, colon-rectum and stomach. World-standardised incidence and mortality rates by sex and period of diagnosis are presented in Tables 1 and 2. The age-standardized rates (ASR) for incidence declined from 308.7 to 289.8 per 100,000 men and from 165.1 to 171.0 per 100,000 women.

**Table 1:** World-standardised cancer incidence and mortality rates in men by tumour site, Martinique, 2001–2015

Period	All cancers		Prostate			Colorectum			Stomach			Lung and bronchial		
	Cases	ASR (W)	Cases	% of all cancers in men	ASR (W)	Cases	% of all cancers in men	ASR (W)	Cases	% of all cancers in men	ASR (W)	Cases	% of all cancers in men	ASR (W)
Incidence														
2001–2005	4155	308.7	2404	57.9%	173.1	224	5.4%	17	217	5.2%	15.4	157	3.8%	12.0
2006–2010	4828	323.6	2676	55.4%	175.9	400	8.3%	26	228	4.7%	13.9	164	3.4%	11.3
2011–2015	4880	289.8	2764	56.6%	162.9	493	10.1%	27.3	226	4.6%	11.7	180	3.7%	10.2
Mortality														
2001–2005	1764	119.6	499	28.3%	28.4	103	5.8%	7.2	164	9.3%	11.1	148	8.4%	11.1
2006–2010	1964	112.1	553	28.2%	25.7	154	7.8%	9.0	161	8.2%	9.4	182	9.3%	11.5
2011–2015^a^	1674	102.0	432	25.8%	20.9	179	10.7%	11.7	154	9.2%	9.3	154	9.2%	10.2

*ASR* Age-standardised rate, *W* world

^a^Mortality data for 2012 not available

**Table 2:** World-standardised cancer incidence and mortality rates in women by tumour site, Martinique, 2001–2015

Period	All cancers		Breast			Colorectum			Stomach			Lung and bronchial		
	Cases	ASR (W)	Cases	% of all cancers in women	ASR (W)	Cases	% of all cancers in women	ASR (W)	Cases	% of all cancers in women	ASR (W)	Cases	% of all cancers in women	ASR (W)
Incidence														
2001–2005	2652	165.1	822	31.0%	55.7	310	11.7%	17.3	164	6.2%	7.6	84	3.2%	5.1
2006–2010	3012	168.1	922	30.6%	56.8	421	14.0%	21.9	193	6.4%	8.0	125	4.2%	6.5
2011–2015	3274	171.0	1129	34.5%	65.9	442	13.5%	19.6	160	4.9%	6.1	152	4.6%	7.0
Mortality														
2001–2005	1339	69.6	220	16.3%	12.9	140	10.4%	6.9	120	8.9%	5.0	60	4.4%	3.5
2006–2010	1532	68.5	222	14.5%	11.1	187	12.2%	7.7	108	7.0%	4.0	134	8.7%	6.1
2011–2015^a^	1413	69.8	231	16.3%	14.4	188	13.2%	8.8	96	6.8%	3.7	97	6.8%	4.9

*ASR* Age-standardised rate, *W* world

^a^Mortality data for 2012 not available

A decline of − 1.05% per year occurred in men during the period 2001–2015 (95% CI [− 1.43; − 0.66]). The age-standardized rates (ASR) for mortality declined from 119.7 to 102.2 per 100,000 men and from 69.6 to 69.8 per 100,000 women. A decline of − 1.46% per year occurred in men during the period 2001–2015 (95% CI [− 2.06; − 0.85]).

### Prostate cancer

Over the period 2011–2015, 4880 new cancer cases were diagnosed in men, including 2764 cases of PCa (56.6% of cancers in men). Prostate cancer, with a mean of 553 registered cases per year and a world-standardised rate of 162.9 per 100,000, is the leading cancer in men; the cumulative rate up to the age of 74 years was 22.8%. A decline of − 1.32% per year occurred during the period 2001–2015 (95% CI [− 1.82; − 0.81]).

**Fig. 2:**
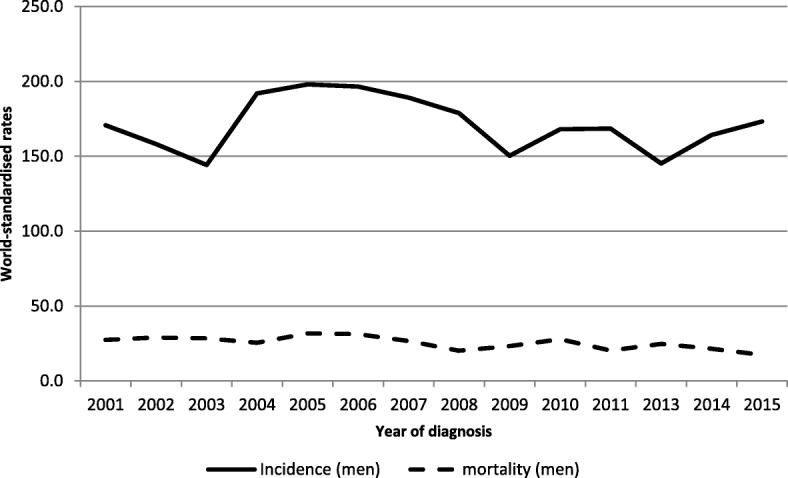
Incidence and mortality world-standardised rates, by year of diagnosis, in prostate cancers, Martinique, 2001–2015. Footnotes: Significant ARC for incidence by 5-year period in men: 5.54 (2001–2005); − 5.74 (2006–2010).

Figure 2 presents world-standardised incidence and mortality rates in prostate cancer.

In terms of mortality, 108 deaths per year were attributable to PCa (25.8% of deaths in men) during the latest period. A decline of − 2.98% per year occurred during the period 2001–2015 (95% CI [− 4.11; − 1.84]).

### Female breast cancer

**Fig. 3:**
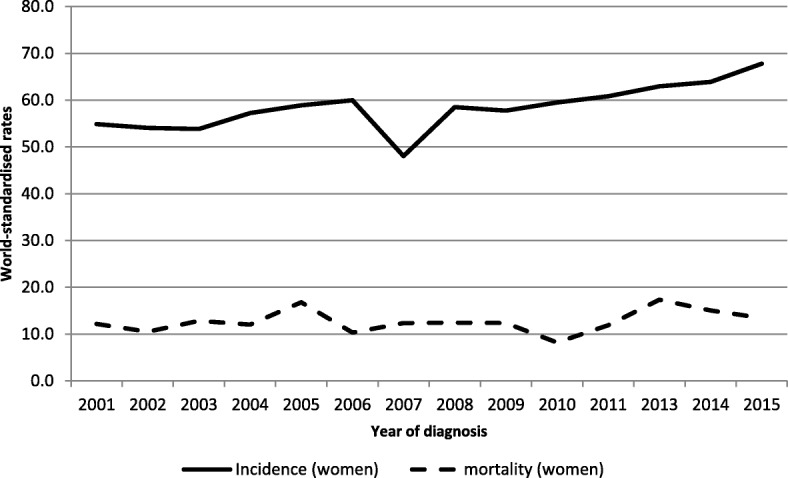
Incidence and mortality world-standardised rates, by year of diagnosis, in breast cancers, Martinique, 2001–2015.

Over the period 2011–2015, 3274 new cases of cancer were diagnosed in women, including 1129 cases of breast cancer (34.5% of cancers in women) – Fig. 3. The cumulative rate up to the age of 74 years was 7.2%. The ARC increased significantly over the period 2001–2015 and 2006–2015 respectively by 1.6% per year (95% CI [0.75, 2.49]) and 2.4% per year (95% CI [0.84; 3.93]). With 226 new cancer cases per year and 58 deaths per year, this cancer is the first tumor site in women in terms of incidence and mortality.

### Colorectal cancer

**Fig. 4:**
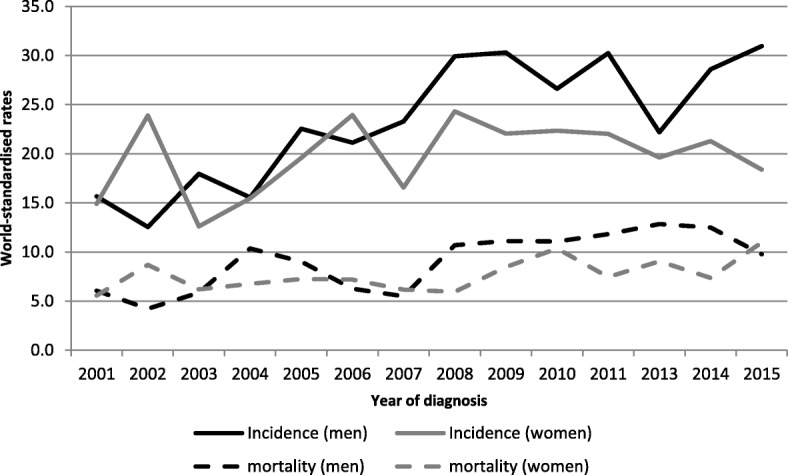
Incidence and mortality world-standardised rates, by year of diagnosis, in colon cancers, Martinique, 2001–2015. Footnotes: Significant ARC for incidence by 5-year period in men: 11.40 (2001–2005).

Colorectal cancer was the second most frequent cancer in men and women, with respectively 99 new cases per year and 45 deaths per year in men and 88 new cases and 47 deaths per year – Fig. 4. The cumulative rate up to the age of 74 years was 3.2% in men and 2.2% in women. Over the period 2001–2015, the ARC increased significantly in men by 4.8% per year (95% CI [3.32, 6.25]). In terms of mortality, the ARC also significantly increased in men and in women between 2001 and 2015 respectively by 4.9% per year (95% CI [2.63; 7.22]) and 2.7% per year (95% CI [0.66; 4.76]).

### Stomach cancer

**Fig. 5:**
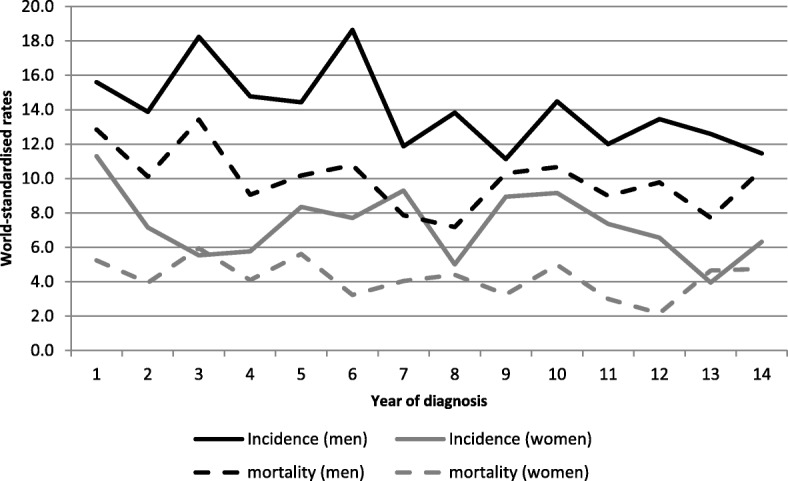
Incidence and mortality world-standardised rates, by year of diagnosis, in stomach cancers, Martinique, 2001–2015.

Stomach cancer was the third tumor site, with 45 new cases per year and 39 deaths per year in men; a total of 32 new cases per year and 24 deaths per year occurred in women – Fig. 5. The cumulative rate up to the age of 74 years was 1.3% in men and 0.6% in women. In men, the ARC decreased in men significantly over the period 2001–2015 (− 2.4% per year 95% CI [− 4.04, − 0.62]). The ARC also decreased significantly in women over the same periods with respectively − 2.9% per year (95% CI [− 4.85; − 0.97]).

### Lung and bronchial cancer

**Fig. 6:**
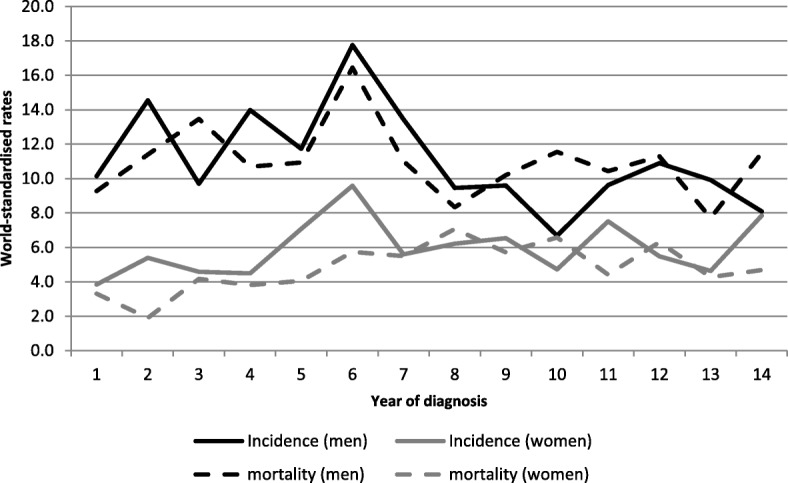
Incidence and mortality world-standardised rates, by year of diagnosis, in lung and bronchial cancers, Martinique, 2001–2015. Footnotes: Significant ARC for incidence by 5-year period in men: − 20.50 (2006–2010). ARC in women: − 12.70 (2006–2010).

Lung and Bronchial cancer was the fourth tumor site, with 36 new cases per year and 39 deaths per year in men; a total of 30 new cases per year and 24 deaths per year occurred in women – Fig. 6. The cumulative rate up to the age of 74 years was 1.2% in men and 0.9% in women. In men, the ARC decreased significantly only over the period 2006–2010 (− 20.5% per year, 95% CI [− 28.9; − 11.2]). In women, the ARC decreased significantly during the same period, by − 12.7% per year (95% CI [− 23.0; 1.12]).

## Discussion

This study provides updated estimates of the cancer burden in Martinique for the study period 2001–2015. Our main results showed a higher incidence of PCa in Martinique (162.9/100,000 men), compared to data from other PBCRs in the Caribbean region [2]. PCa incidence and mortality rates are high in the Caribbean (respectively 79.8 and 29.0 cases per 100,000 men); the French West-Indies rank first for both PCa incidence and mortality rates compared to Metropolitan France (world-standardised incidence rate of 97.7/100,000 and mortality rate of 10.5/100,000 in 2012 in Metropolitan France) [2].

According to mortality data from the *CépiDC* for the period 2013–2015 in Martinique, cancer is the leading cause of death for both sexes combined (26.5%), ahead of diseases of the circulatory system (24.5%). In men, cancer mortality was 28.3%, versus 22.5% for diseases of the circulatory system. In women, cancer remains in second place at 24.7%, behind diseases of the circulatory system, which account for 26.5%. In total, over the last period (2011–2015), 3087 deaths occurred, corresponding to an average of 772 deaths per year (Table 1).

From January 2008 to December 2013, a total of 3295 cases of PCa were analysed by the population-based cancer registry of Guadeloupe. The PCa incidence was almost twice as high as that in Metropolitan France (98.0 cases per 100,000 men) with world-standardized incidence and mortality of 184.1 [177.8–190.4] and 23.9 [21.9–25.7] per 100,000 person-years [11].

The Cancer Incidence in Five Continents Volume XI published by the International Agency for Research on Cancer and the International Association of Cancer Registries provides statistics on the incidence of cancer from cancer registries around the world between 2008 and 2012. PCa incidence in French Guyana for this period was 118.5/100,000 men [12]. High rates were also observed in Barbados (123.1/100,000) and Trinidad and Tobago (123.9/100,000) according to GLOBOCAN 2012 estimations [3].

The introduction of prostate-specific antigen (PSA) testing for PCa, has led to an increase of PCa cases in recent years. Among the risk factors for PCa, family history and genetic factors are well known [13], but other factors such as environmental exposures or lifestyle need to be explored. Environmental exposure to chlordecone, an insecticide and fungicide intensively applied to banana plantations from 1973 to 1993 in the French West-Indies, may have increased PCa incidence in this region [14, 15]. A population-based case–control study was carried out in Guadeloupe from 2005 to 2007 to investigate the relationship between exposure to chlordecone and the risk of PCa [14]; a significant positive association between chlordecone exposure and PCa risk was confirmed (OR 1.65, 95%CI 1.09, 2.48; *p* value for trend = 0.01) [16].

The most frequent cancers in the Caribbean in both sexes are prostate, breast, lung and bronchial, colorectal, and cervical cancers, while lung and bronchial cancers, prostate, colorectal, breast and stomach are five most frequent sites for cancer deaths [2].

Multicentre observational studies will help to improve our understanding of the burden of cancer in this area. A first comparative analysis was performed on data from the cancer registries of Guadeloupe, French Guyana, and Martinique with regard to invasive tumours of the stomach, colon-rectum, prostate and breast. This study reported similar cancer profiles between Martinique and Guadeloupe, with higher incidence rates of PCa [17].

However, data are sparse regarding cancer in the populations of the Caribbean/Central America [18]. Research papers have been published by the oncology department and the MCR mainly on PCa, lung cancer and colorectal cancer [19–26]. Data from Caribbean population-based cancer registries will make it possible to identify clinical and epidemiological characteristics of cancer and to study survival [11, 23, 24, 26–28].

Leading causes of cancer mortality were analysed for the Caribbean Region during 2003–2013. Prostate cancer and lung cancer were the most frequent causes of deaths, with respectively 18.4 to 47.4% of cases and 5.6 to 24.4% of cases in men. Among women, breast (14.0 to 29.7%) and cervical (4.5 to 18.2%) cancers were the most frequent causes of deaths [29] .

Cancer is also the leading cause of death in Cuba, with 24.9% of deaths due to cancer in 2013 (22,982 /92,273) [30]; lung and bronchial, prostate, breast and colon cancers were the main causes of cancer deaths. With 44,608 new cancer cases in 2013, the highest rates of cancer incidence reported by the Cuba population-base cancer registry correspond to skin cancer, prostate, breast, lung and bronchial, cervix. The data from Cuba included non-melanoma skin cancers, which are typically excluded from most cancer data reports [30].

In Puerto-Rico, 15,392 new cases of cancer were reported in 2012, while 5437 people died from cancer. Prostate and breast cancer were the most commonly diagnosed cancers, and the leading causes of cancer death. Colorectal cancer was the second most diagnosed cancer in both men and women. Lung cancer was the second leading cause of cancer death in men, and ranked third among causes of cancer death in women. Conversely, cancer of the colon and rectum was the third leading cause of cancer deaths among men and the second leading cause of cancer deaths in women [31].

In women, breast cancer ranks first in Martinique in terms of incidence (65.9/100,000 women) and mortality (14.4/100,000 women). The incidence is higher than that observed in the rest of the Caribbean, but remains lower than that observed in Metropolitan France in 2012 (world-standardised incidence rate of 88.0/100,000 and mortality of 15.7/100,000 women in Metropolitan France). The development of screening programmes for women aged between 50 and 74 years has driven the increase in cancer incidence observed in Martinique. The main findings indicate that Martinique breast cancer incidence increased in the last period, and this result can likely be explained by the screening programme started in Martinique in 2004, and the development of mammography and echography practices on the island. Results of a population-based study in Guadeloupe did not report a pattern of more aggressive breast cancer in the age group preceding that eligible for mass screening, despite a lower age of cancer diagnosis in this population [27, 28].

Colorectal cancer ranks as the second most frequent cancer in both men and women in Martinique and Guadeloupe, and 4th and 3rd most frequent in men and women respectively in French Guyana [17]. With an incidence rate considerably higher than that observed in South America, but contrary to PCa, incidence rates for colorectal cancer are lower in the French West Indies compared to Metropolitan France [32]. Few data are available regarding colorectal cancer in the French West Indies [20, 23, 24]; a first study from the MCR confirmed that incidence of colorectal cancer started to increase in the 2000s. The trends observed reflect a salient epidemiological transition in the Caribbean [23].

Gastric cancer was also among first sites of cancer incidence and mortality in Martinique. There remains an unmet need to control Helicobacter pylori infection and other risk factors, as well as to improve diagnosis and management, to further reduce the burden of gastric cancer in the Caribbean. The decrease observed in gastric cancer can be explained by the decline in the prevalence of Helicobacter pylori infection, and by improvements in food preservation and diet [33]. Yet, the burden of gastric cancer still remains very high in several countries from Latin America and the Caribbean [32].

Among women, the increase in lung cancer incidence observed is mainly due to changes in smoking habits in women. In Martinique, lung cancer incidence rates are lower than those reported in metropolitan France in 2012 (51.7/100,000 men and 18.6/100,000 women in Metropolitan France). Mortality rates are also low compared to national data (37.0/100,000 men and 12.9/100,000 women in Metropolitan France) [34]. This highlights the changing difference between men and women, with the gap between the sexes expected to decrease as the number of female smokers increases. Other studies were also performed on genetic factors of lung cancer in Martinique; 157 patients were studied in Martinique and very high levels of EGFR mutation were found, contrary to what is found in in Metropolitan France or in African Americans [35].

Recording cancer data in cancer registries is essential for the production of reliable epidemiological data, and also contributes to improving management and reducing mortality. It would therefore be beneficial if other, existing registries could contribute to the production of international epidemiological statistics for this area [36, 37].

The IARC Caribbean Cancer Registry Hub was launched in 2018 *to improve the availability, use and dissemination of high-quality cancer data to inform cancer control in the Caribbean region* [38]; this hub will respond to the need for better cancer surveillance data in the island countries of the Caribbean. IARC has shared news with Caribbean collaborators that they are developing an agreement with Martinique for work in the Caribbean, notably the French zone.

The collaboration that is undertaken will make it possible to produce comparative data, and to work on research topics such as cancer inequalities across the Caribbean, surveillance of infectious cancers, the role of environmental factors and specific exposures in our geographical area, or identification of the factors that determine health states in patients suffering from cancer in the Caribbean zone.

## Conclusions

Data regarding the epidemiology of certain types of cancer in Martinique were scarce and unreliable, and therefore, the MCR was created to enable exhaustive and continuous recording of all cases of cancer in persons living in Martinique.

Prostate cancer and stomach cancers have a higher incidence in Martinique and rank first among the four major tumor sites. These cancers need to be analysed in further epidemiological studies to explore patterns of care among patients via population-based studies and genomic studies.

## Abbreviations

ARC: Annual rate of change
ASR: Age-standardized rates
CI: Confidence interval
ENCR: European Network of Cancer Registries
MCR: Martinique Cancer Registry
PBCR: Population-based cancer registries
PCa: Prostate cancer
PSA: Prostate-specific antigen
